# Diagnostic Predictive Model for Distinguishing Intravascular Large B-Cell Lymphoma Among Patients with Fever of Unknown Origin

**DOI:** 10.3390/diagnostics16172768

**Published:** 2026-08-28

**Authors:** Min Lang, Chao Chen, Yiao Di, Zhe Zhuang, Zepeng Li, Congwei Jia, Ximin Shi, Danqing Zhao, Wei Wang, Wei Zhang, Daobin Zhou, Yan Zhang

**Affiliations:** 1 Department of Hematology, Peking Union Medical College Hospital, Chinese Academy of Medical Sciences & Peking Union Medical College, No.1 Shuaifuyuan Wangfujing Dongcheng District, Beijing 100730, China; 2 Department of Laboratory Medicine, Peking Union Medical College Hospital, Chinese Academy of Medical Sciences & Peking Union Medical College, Beijing 100730, China; 3 Department of Pathology, Peking Union Medical College Hospital, Chinese Academy of Medical Sciences & Peking Union Medical College, Beijing 100730, China; 4 Department of Nuclear Medicine, Peking Union Medical College Hospital, Chinese Academy of Medical Sciences & Peking Union Medical College, Beijing 100730, China

**Keywords:** intravascular large B-cell lymphoma, logistic regression, fever of unknown origin

## Abstract

**Background/Objectives**: Intravascular large B-cell lymphoma (IVLBCL) is a rare and diagnostically challenging disease, often presenting as fever of unknown origin (FUO). This study aimed to develop and validate diagnostic predictive models and scoring systems to distinguish IVLBCL from other causes of FUO in hospitalized patients. **Methods**: A retrospective analysis was conducted in patients with IVLBCL or other causes of FUO who were treated between February 2015 and October 2023. Two multivariable logistic regression models and corresponding integer-based scoring systems were developed in a training cohort comprising 42 patients with IVLBCL and 45 FUO controls. Internal validation was performed using leave-one-out cross-validation and bootstrap resampling, followed by temporal validation in an independent cohort of 18 patients with IVLBCL and 21 FUO controls. Model 1 was additionally evaluated for sensitivity in an external case-only cohort comprising 40 patients with IVLBCL from eight hospitals. **Results**: Model 1 incorporated peripheral edema, hypoxemia, neurological symptoms, hemophagocytic lymphohistiocytosis, and interstitial lung abnormalities on computed tomography and achieved an area under the receiver operating characteristic curve (AUC) of 0.916 in the training cohort. Model 2 combined the interleukin-10/interleukin-6 (IL-10/IL-6) ratio with peripheral edema, hypoxemia, and neurological symptoms and demonstrated significantly improved discrimination (AUC = 0.982, *p* = 0.021 vs. Model 1). In the temporal validation cohort, the AUCs of Models 1 and 2 were 0.975 and 0.997, respectively. The corresponding integer-based scoring systems achieved AUCs of 0.903 and 0.952 in the training cohort and 0.926 and 0.992 in the temporal validation cohort. In the external case-only cohort, both Model 1 and its integer-based score identified 32 of 40 patients, yielding a sensitivity of 80.0%. Random skin biopsy provided the histological diagnosis in 58% of cases, with a positivity rate of 79.5%. **Conclusions**: Two diagnostic models and their simplified scoring systems were developed and internally and temporally validated to aid the diagnosis of IVLBCL in hospitalized patients with FUO. These models may assist in the diagnostic workup of hospitalized FUO patients, especially when IL-10/IL-6 testing is unavailable. Their performance in outpatient or community settings remains uncertain, and prospective multicenter validation with appropriate FUO controls is warranted.

## 1. Introduction

Intravascular large B-cell lymphoma (IVLBCL) is a rare and distinct subtype of extranodal diffuse large B-cell lymphoma, characterized by the selective proliferation of malignant B cells within the lumina of small blood vessels, particularly capillaries [1]. As the neoplastic cells typically do not circulate in peripheral blood and do not form macroscopic tumor masses, the clinical presentation is highly heterogeneous [2,3].

In Asian populations, fever of unknown origin (FUO), defined as fever ≥ 38.3 °C on several occasions, lasting more than three weeks, without an established cause despite appropriate investigation [4,5], is the most common systemic manifestation, reported in 87–100% of patients, and is frequently the presenting symptom [6,7,8]. Other clinical features reflect the sites of vascular involvement; the central nervous system, skin, and lungs are most commonly affected, giving rise to diverse neurological deficits, cutaneous lesions, and respiratory symptoms [9,10,11]. In addition, some patients develop hemophagocytic lymphohistiocytosis (HLH) or present with peripheral edema [12,13,14].

The diagnosis of IVLBCL remains challenging and requires histological confirmation. The nonspecific and protean nature of the symptoms often leads to substantial diagnostic delays. Notably, retrospective series from Japan and Western countries with over a decade of follow-up have reported that 16–24% of IVLBCL cases were diagnosed only at autopsy, underscoring the difficulty of timely recognition during life [15]. Given that IVLBCL is an aggressive lymphoma with a dismal prognosis when diagnosis is delayed, there is a pressing need for strategies that enable earlier clinical suspicion and targeted diagnostic workup.

We previously identified interleukin-10 (IL-10) as a valuable biomarker for the early diagnosis of IVLBCL [16]. Since the majority of patients with IVLBCL present with FUO, this study aimed to construct and validate diagnostic predictive models that could distinguish IVLBCL from other causes of FUO using readily available clinical features and the interleukin-10/interleukin-6 (IL-10/IL-6) ratio. We further evaluated the diagnostic yield of various biopsy methods and sought to propose a practical diagnostic algorithm to facilitate the timely identification of IVLBCL.

## 2. Materials and Methods

### 2.1. Study Design and Participants

This retrospective cohort study was conducted at Peking Union Medical College Hospital between February 2015 and October 2023, and was approved by the Institutional Review Board of Peking Union Medical College Hospital (approval no. K3426; approved on 9 January 2025). The study was performed in accordance with the Declaration of Helsinki. All patients provided informed consent. A waiver was granted from the ethics committees of the eight participating external hospitals. A total of 145 patients were initially identified from the database. After excluding 12 patients with incomplete clinical data and 7 patients with alternative definite diagnoses, 60 patients with IVLBCL and 66 patients with FUO were enrolled and assigned to a training cohort (42 IVLBCL and 45 FUO, diagnosed between February 2015 and October 2021) and a temporal validation cohort (18 IVLBCL and 21 FUO, diagnosed between November 2021 and October 2023). Inclusion criteria for IVLBCL patients were: (i) FUO as a presenting feature and (ii) histologically confirmed IVLBCL. Inclusion criteria for FUO patients were: (i) unexplained fever persisting for >3 weeks and (ii) definite exclusion of IVLBCL. An independent external case-only sensitivity evaluation cohort was retrospectively collected from eight external hospitals between February 2014 and December 2025. Eligible patients had histologically confirmed IVLBCL with FUO as the initial symptom.

### 2.2. Data Collection and Definitions

Demographic data, clinical characteristics (including neurological symptoms and physical findings), and laboratory results (hematological, biochemical, and immunological) were obtained from electronic medical records. Pre-treatment chest computed tomography (CT) images were reviewed, and pathological records of all biopsy specimens (skin, lung, and bone marrow) were collected. Random skin biopsies were performed by the dermatology department; specimens were typically obtained from three separate, fat-containing skin sites.

For the purpose of this study, the following definitions were adopted: peripheral edema was defined as an abnormal accumulation of fluid in peripheral tissues, typically in the extremities; hypoxemia was defined as an arterial oxygen partial pressure (PaO_2_) < 60 mmHg on room air; skin rash encompassed redness, itching, inflammation, blistering, or other visible skin alterations; hemophagocytic lymphohistiocytosis (HLH) was diagnosed according to the HLH-2004 criteria [17]; and interstitial lung abnormalities (ILA) were identified on CT as ground-glass or reticular abnormalities, lung distortion, traction bronchiectasis, honeycombing, or non-emphysematous cysts [18].

### 2.3. Statistical Analysis

**Figure 1:**
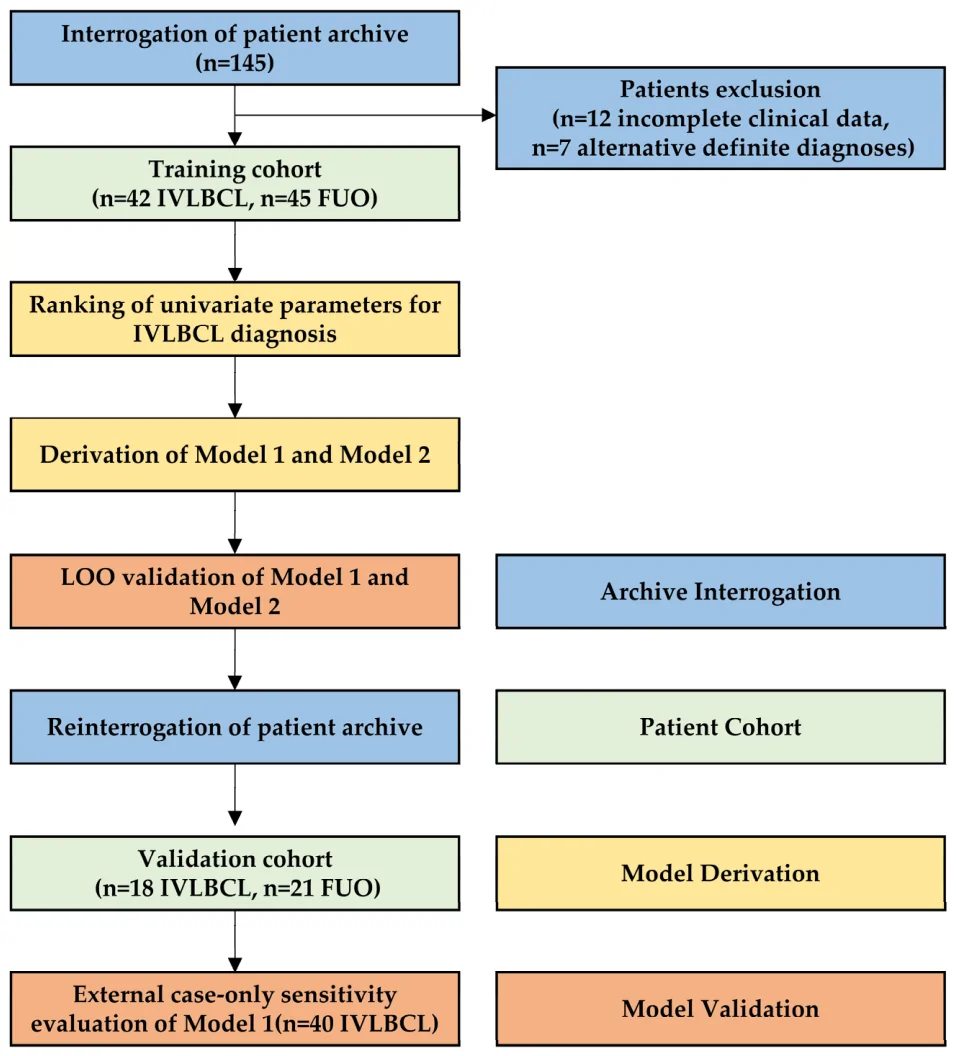
Flowchart outlining the study.

The overall study flow is depicted in Figure 1. The reporting of this prediction model study follows the TRIPOD (Transparent Reporting of a multivariable prediction model for Individual Prognosis or Diagnosis) statement [19]. All statistical analyses were performed using SPSS version 27.0 (SPSS Inc., Chicago, IL, USA) and R version 3.6.3 (R Foundation for Statistical Computing, Vienna, Austria). The R analysis code is provided as a supplementary script (Supplementary File S1). Continuous variables were tested for normality; the Mann–Whitney U test was used for non-normally distributed variables, the independent samples *t*-test for normally distributed variables with equal variances, and the chi-square test (with Fisher’s exact test where appropriate) for categorical variables.

At our center, the detection range for IL-10 and IL-6 was 5–1000 pg/mL. For the calculation of the IL-10, values below 5 pg/mL were set to 0, and values above 1000 pg/mL were truncated to 1000 pg/mL; for the calculation of the IL-6, values below 5 pg/mL were set to 5, and values above 1000 pg/mL were truncated to 1000 pg/mL. Because the ratio was highly skewed, it was also evaluated as a binary variable using the optimal cut-off of 0.450 derived from ROC analysis. The continuous ratio was used in Model 2, whereas the binary ratio (≥0.450) was used to construct the Model 2 scoring system. The area under the receiver operating characteristic (ROC) curve (AUC) was calculated to assess the discriminatory ability of the IL-10/IL-6 ratio. Candidate predictors were selected based on clinical relevance and univariable association with IVLBCL at *p* < 0.10. The final multivariable models included variables that were clinically important and had sufficient event frequency to avoid sparse-data bias. Two diagnostic models were constructed using multivariable logistic regression to distinguish IVLBCL from FUO. Model 1 comprised clinical and imaging variables only. Model 2 was constructed as a separate model combining the IL-10/IL-6 ratio with three clinical variables (peripheral edema, hypoxemia, and neurological symptoms); Model 2 is not strictly nested within Model 1 because Model 1 additionally includes HLH and ILA. Simplified integer-based scoring systems were subsequently derived from the final models: each binary predictor in Model 1 and Model 2 was assigned one point, and the IL-10/IL-6 ratio was categorized at its optimal cut-off (≥0.450 = 1 point) (details are provided in Section 3).

Multicollinearity was assessed using variance inflation factors (VIFs); all VIFs were <2.5 (Table S1) [20]. To account for potential overfitting in the small sample, we additionally fitted Firth penalized logistic regression models and compared the results with standard logistic regression (Table S2) [21]. The DeLong test was used to compare the AUCs of the models and the IL-10/IL-6 ratio [22]. The incremental value of the IL-10/IL-6 ratio over clinical variables was assessed by comparing Model 1 with Model 1 plus the IL-10/IL-6 ratio (a nested comparison), using the DeLong test and the likelihood ratio test; the incremental value of clinical variables over the ratio was assessed by comparing Model 2 with the ratio alone, using the DeLong test.

Internal validation was performed by leave-one-out cross-validation and bootstrap internal validation with 1000 resamples to obtain optimism-corrected AUCs [23]. In leave-one-out cross-validation, predicted probabilities from all out-of-fold observations were pooled to compute a single ROC curve and AUC; the Brier score was calculated as the mean squared difference between predicted probabilities and observed outcomes. The resulting models and the ratio were then applied to the temporal validation cohort to assess their diagnostic performance.

For the external case-only sensitivity evaluation cohort, Model 1 and its integer-based scoring system were applied using the fixed cut-offs established in the training cohort. Sensitivity was calculated as the proportion of IVLBCL patients correctly classified as positive; specificity and AUC could not be computed because the cohort contained only confirmed IVLBCL cases without FUO controls.

## 3. Results

### 3.1. Clinical Characteristics

The baseline characteristics of the training cohort (42 IVLBCL, 45 FUO) are summarized in Table 1. Median age (58.5 vs. 56 years, *p* = 0.074) and sex distribution (male, 54.8% vs. 51.1%, *p* = 0.831) were comparable. Peripheral edema, hypoxemia, neurological symptoms, and HLH were significantly more frequent in IVLBCL patients. The most common neurological symptoms among IVLBCL patients were extremity weakness (11, 26.2%) and cognitive impairment (6, 14.3%). ILA on CT were also more prevalent in IVLBCL patients. Serum IL-10 concentration and the IL-10/IL-6 ratio were significantly higher in IVLBCL patients than in FUO patients. Among the 45 FUO patients, final diagnoses included infectious disease (n = 14), connective tissue disease (n = 13), hematological disease (n = 10), malignancy (n = 5), and other causes (n = 3).

### 3.2. Diagnostic Model Generation

**Figure 2:**
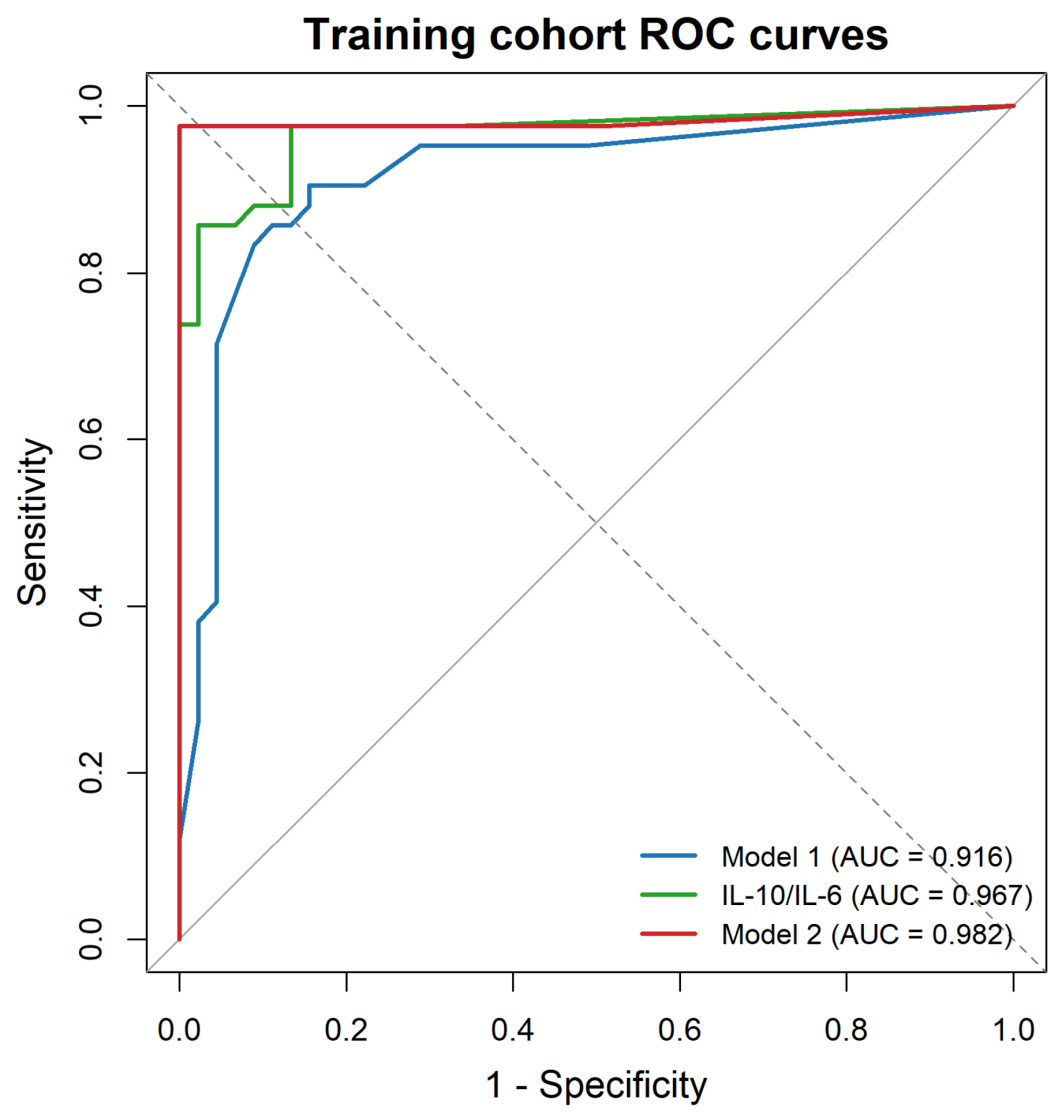
The ROC curves of IL-10/IL-6, Model 1 and Model 2 applied to the training cohort. The areas under the ROC curves for the ability to differentiate IVLBCL from FUO for Model 1, IL-10/IL-6, and Model 2 were 0.916, 0.967 and 0.982, respectively.

Two multivariable logistic regression models were constructed (Table 2). Model 1 included five clinical variables: peripheral edema, hypoxemia, neurological symptoms, HLH, and ILA. In Model 1, variables associated with IVLBCL were peripheral edema (OR 5.348, 95% CI 1.011–32.393, *p* = 0.053) and hypoxemia (OR 13.354, 95% CI 3.041–74.551, *p* = 0.001). The IL-10/IL-6 ratio demonstrated excellent discriminatory ability in the training cohort, with an optimal cut-off of 0.450 (AUC 0.967, sensitivity = 97.6%, specificity = 86.7%). Model 2 was constructed as a separate model combining the IL-10/IL-6 ratio with three clinical variables (peripheral edema, hypoxemia, and neurological symptoms). In Model 2, hypoxemia (OR 31.504, 95% CI 2.046–1797.170, *p* = 0.029) and the IL-10/IL-6 ratio (OR 9.195, 95% CI 2.619–70.708, *p* = 0.006) were significant predictors. Model 1 and Model 2 achieved AUCs of 0.916 and 0.982, respectively (Table 3, Figure 2). The AUC of Model 2 was significantly higher than that of Model 1 (DeLong test, *p* = 0.021).

Because the models are not nested, this comparison should be interpreted with caution. Multicollinearity was assessed using variance inflation factors (VIFs) (Table S1). To account for potential overfitting in the small sample, Firth penalized logistic regression was performed (Table S2): for Model 1, hypoxemia (OR 9.907, 95% CI 2.47–39.735, *p* < 0.001) was a significant predictor; for Model 2, hypoxemia (OR 8.076, 95% CI 1.598–40.805, *p* = 0.016) and the IL-10/IL-6 ratio (OR 1.495, 95% CI 1.160–1.926, *p* < 0.001) were significant predictors. Incremental value analysis in the training cohort showed that adding the IL-10/IL-6 ratio to Model 1 significantly improved discrimination (DeLong test, *p* = 0.020; likelihood ratio test *p* < 0.001). Conversely, adding the clinical variables to the ratio (i.e., comparing Model 2 with the IL-10/IL-6 ratio alone) did not significantly improve the AUC (DeLong test, *p* = 0.129).

### 3.3. Internal Validation

Leave-one-out cross-validation was performed by pooling all out-of-fold predicted probabilities into a single set and computing one ROC curve and one AUC per model (fold-specific AUCs are undefined because each fold contains a single test patient). The pooled out-of-fold AUCs were 0.959, 0.867, and 0.972, and the corresponding Brier scores were 0.075, 0.125, and 0.041, for the IL-10/IL-6 ratio, Model 1, and Model 2, respectively. Bootstrap internal validation with 1000 resamples yielded optimism-corrected AUCs of 0.893 for Model 1 and 0.976 for Model 2, indicating minimal overfitting.

### 3.4. Temporal Validation

**Figure 3:**
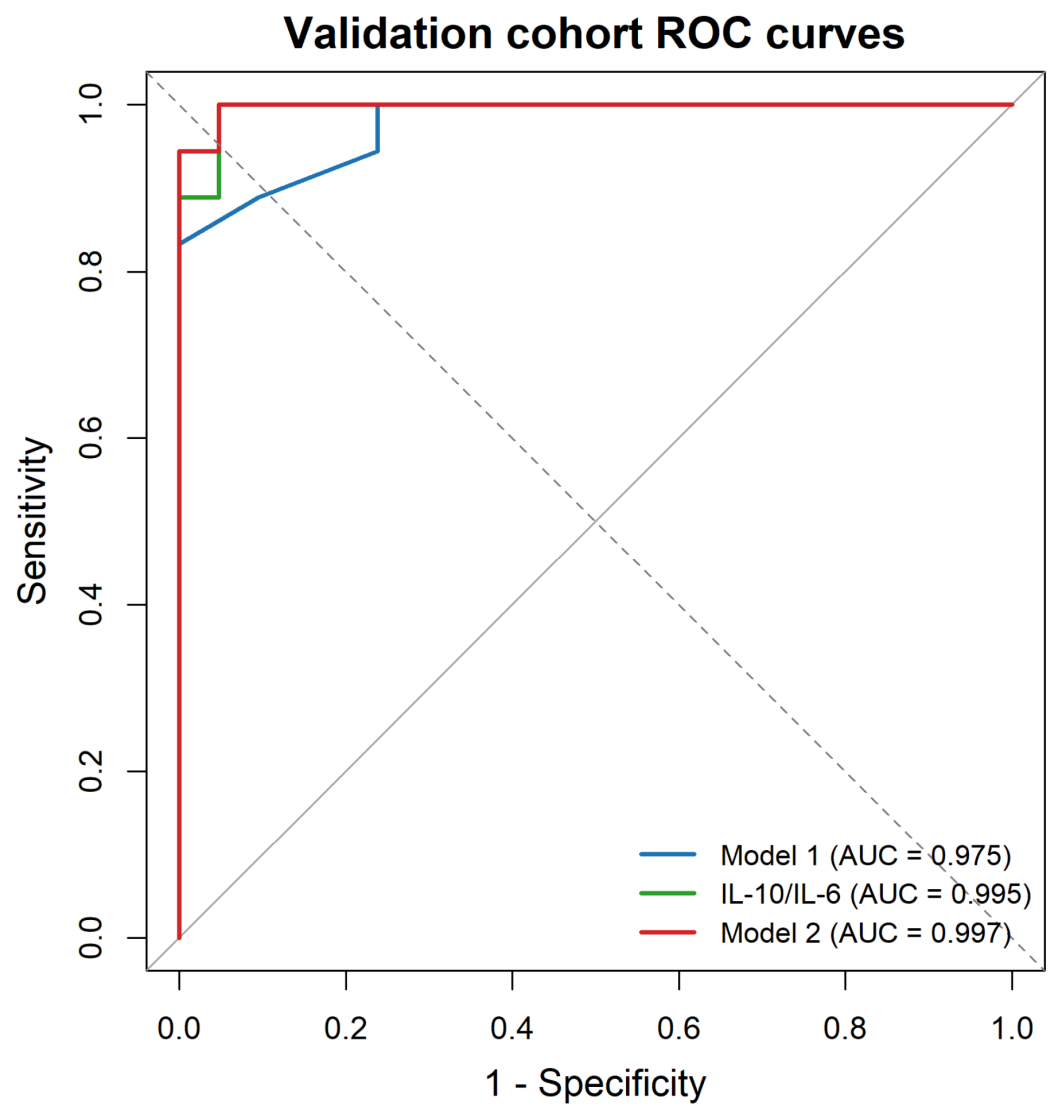
The ROC curves of IL-10/IL-6, Model 1 and Model 2 applied to the validation cohort. The areas under the ROC curves for the ability to differentiate IVLBCL from FUO for Model 1, IL-10/IL-6, and Model 2 were 0.975, 0.995 and 0.997, respectively.

Temporal validation in the independent cohort (18 IVLBCL, 21 FUO) showed that the IL-10/IL-6 ratio maintained an AUC of 0.995 (training AUC 0.967, *p* = 0.156). At the cut-off of 0.450, sensitivity was 100.0% and specificity was 95.2%. Model 1 achieved an AUC of 0.975 (training AUC 0.916, *p* = 0.125), with sensitivity = 88.9% and specificity = 90.5% at a cut-off of 0.296. Model 2 achieved an AUC of 0.997 (training AUC 0.982, *p* = 0.406), with 94.4% sensitivity and 100% specificity at a cut-off of 0.576 (Figure 3). Incremental value analysis in the temporal validation cohort showed that adding the IL-10/IL-6 ratio to Model 1 did not significantly improve discrimination (DeLong test, *p* = 0.242), although the likelihood ratio test was significant (*p* = 0.004). The addition of clinical variables to the ratio (Model 2 vs. ratio alone) did not significantly improve the AUC (DeLong test, *p* = 0.480).

### 3.5. Integer-Based Scoring System

**Figure 4:**
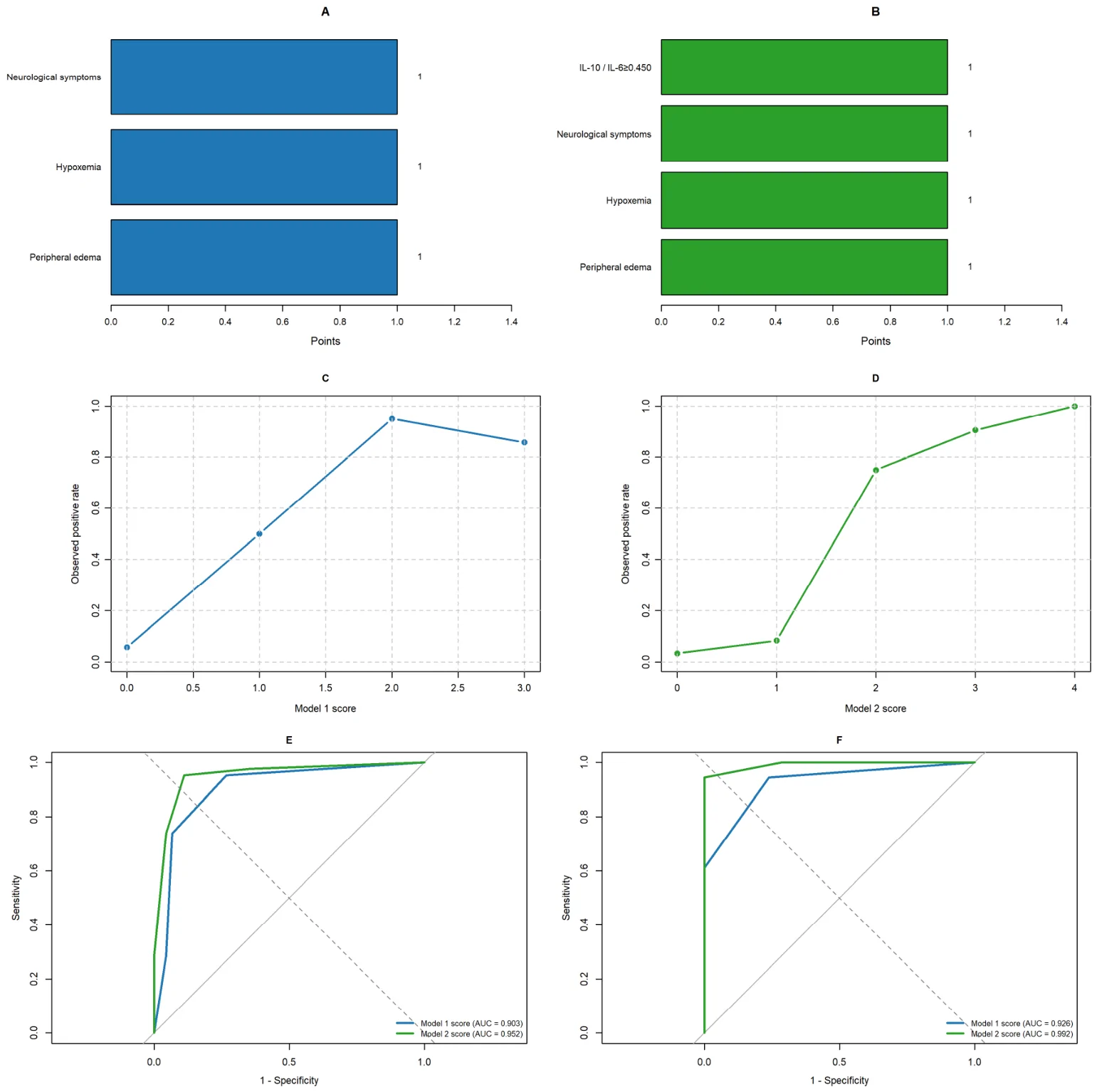
Integer-based scoring systems generated from Model 1 and Model 2. (**A**) Model 1 score composition: peripheral edema, hypoxemia, and neurological symptoms each contribute 1 point if present, yielding a total score ranging from 0 to 3. (**B**) Model 2 score composition: in addition to the three clinical variables, an IL-10/IL-6 ratio ≥ 0.450 contributes 1 point, yielding a total score ranging from 0 to 4. (**C**,**D**) Relationship between integer-based scores and observed positive rates (IVLBCL diagnosis) in the training cohort. (**E**) ROC curves of the integer-based scoring systems applied to the training cohort. (**F**) ROC curves of the integer-based scoring systems applied to the validation cohort.

To facilitate bedside use, simplified integer-based scoring systems were derived from the key clinical predictors (Figure 4A,B): each present predictor contributes 1 point. The Model 1 score (range 0–3) sums peripheral edema, hypoxemia, and neurological symptoms; the Model 2 score (range 0–4) additionally adds 1 point when the IL-10/IL-6 ratio is ≥0.450. The relationships between the integer-based scores and observed positive rates are shown in Figure 4C,D. In the training cohort, the AUCs of the Model 1 and Model 2 scoring systems were 0.903 and 0.952, respectively; using a cut-off of ≥1, the Model 1 score achieved sensitivity 95.2% and specificity 73.3%, and the Model 2 score achieved sensitivity 97.6% and specificity 64.4%. In the validation cohort, the AUCs were 0.926 and 0.992, respectively (Figure 4E,F); using the same cut-off of ≥1, the Model 1 score achieved sensitivity 94.4% and specificity 76.2%, and the Model 2 score achieved sensitivity 100.0% and specificity 71.4%.

### 3.6. Biopsy Confirmation

All 60 IVLBCL patients in the training and validation cohorts were diagnosed by random skin biopsy, bone marrow biopsy, and/or transbronchial lung biopsy (TBLB) (Table 4). Most patients underwent bone marrow biopsy, but the positivity rate was only 42.1%. Random skin biopsy was performed in 44 patients with a positivity rate of 79.5%. TBLB was performed in eight patients, all of whom had hypoxemia and ILA on CT, and all eight were diagnosed by this procedure. Among patients classified as IVLBCL by Model 1 and Model 2, random skin biopsy positivity rates were 80.0% and 81.0%, respectively.

### 3.7. External Case-Only Sensitivity Evaluation

The external cohort comprised 40 histologically confirmed IVLBCL patients with FUO from eight hospitals (Table S3). Median age was 63 years (range 39–84), and 21 (52.5%) were male. Compared with the training cohort, the ex-ternal cohort had lower frequencies of neurological symptoms (25.0% vs. 54.8%, *p* = 0.007) and ILA on CT (30.0% vs. 64.3%, *p* = 0.002). Peripheral edema (67.5% vs. 64.3%, *p* = 0.818), hypoxemia (60.0% vs. 78.6%, *p* = 0.093) and HLH (35.0% vs. 40.5%, *p* = 0.654) were comparable. At the pre-specified cut-off of 0.296, Model 1 correctly identified 32 of 40 patients, yielding a sensitivity of 80.0% (95% CI 64.4–90.9%). At a score cut-off of ≥1, the integer-based Model 1 score identified 32 of 40 patients (sensitivity 80.0%). Biopsy positivity rates were consistent with the derivation findings: random skin biopsy was positive in 6 of 6 patients (100%), TBLB in 2 of 2 (100%), and bone marrow biopsy in 14 of 38 (36.8%).

## 4. Discussion

The diagnosis of IVLBCL remains a formidable clinical challenge, largely due to the absence of circulating neoplastic cells and the non-specific nature of its presentation [12,24]. FUO is the most frequent initial manifestation, especially in Asian populations [6,25]. In this study, we constructed and rigorously validated two practical diagnostic models to distinguish IVLBCL from other causes of FUO in hospitalized patients, and demonstrated that incorporating the IL-10/IL-6 ratio significantly improves diagnostic accuracy over clinical features alone.

Model 1 integrates five readily obtainable clinical and imaging variables that directly reflect the microvascular occlusion and organ involvement characteristic of IVLBCL [12] and were significantly more frequent in IVLBCL patients than in FUO controls. The model achieved an AUC of 0.916 in the training cohort, with robust performance on internal validation (bootstrap-corrected AUC 0.893) and temporal validation (AUC 0.975). In an external cohort where key manifestations were less prevalent, likely reflecting a milder disease spectrum outside a tertiary referral center, Model 1 still identified 80.0% of cases. This finding indicates that Model 1 may help raise suspicion of IVLBCL in selected hospitalized FUO patients, but its sensitivity in less symptomatic or outpatient populations is limited. A positive prediction can prompt random skin biopsy and justify IL-10/IL-6 measurement to enable Model 2 refinement; however, a negative prediction should not be used to rule out IVLBCL. The integer-based scoring system performed similarly, supporting its bedside applicability in hospitalized settings. Whole-body FDG-PET/CT may complement the clinical models by revealing multifocal hypermetabolic lesions suggestive of microvascular involvement, although tracer avidity is heterogeneous across patients [26].

**Figure 5:**
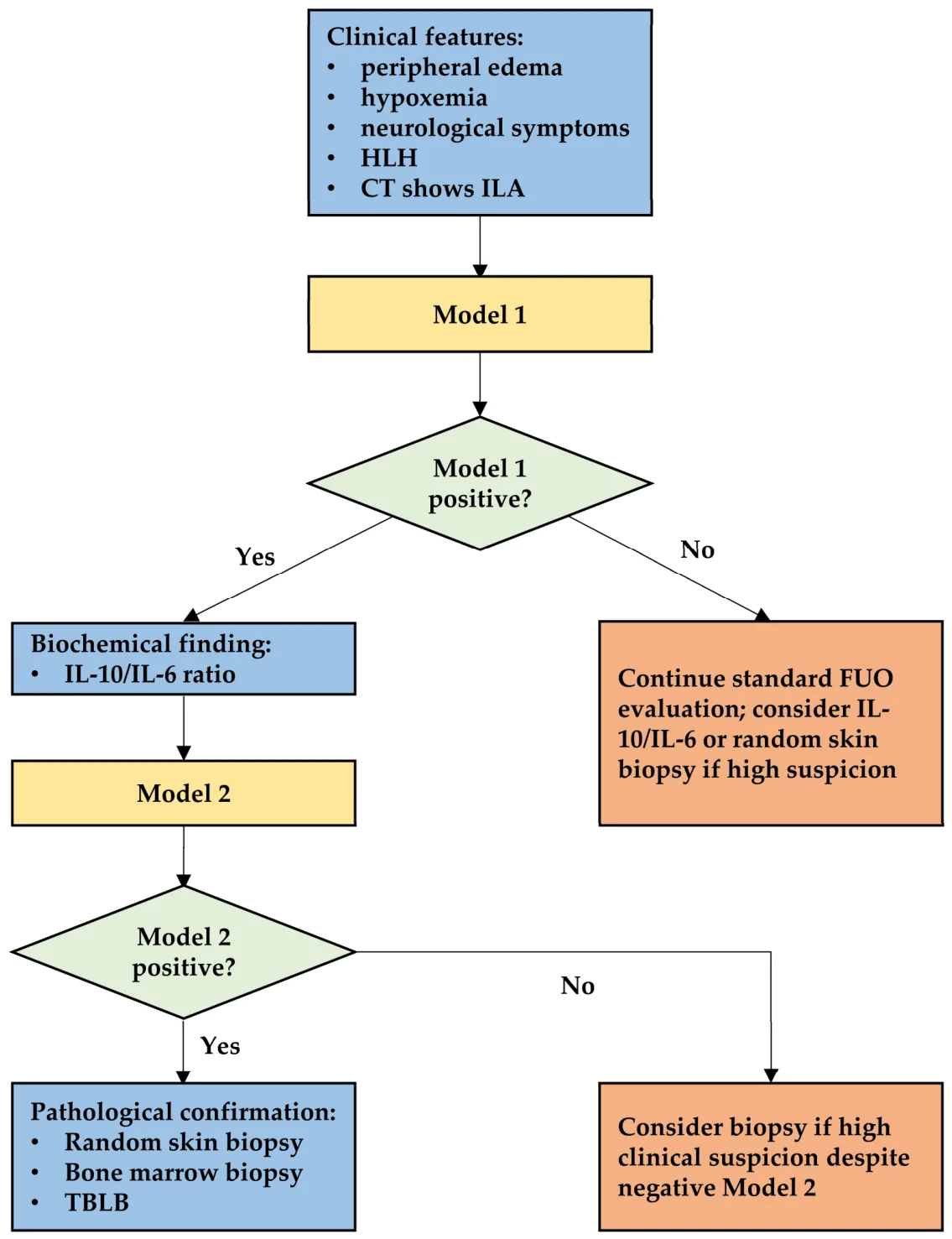
Proposed diagnostic algorithm for IVLBCL in hospitalized patients with FUO. Model 1 is applied first; if positive, serum IL-10/IL-6 is measured and Model 2 is applied. If Model 2 is positive, biopsy (random skin biopsy, bone marrow biopsy, or TBLB) is performed. If either model is negative, standard FUO evaluation should continue, and biopsy should be reconsidered if clinical suspicion remains high.

We have previously shown that serum IL-10 is a valuable biomarker for early IVLBCL diagnosis [16]. In the present analysis, the IL-10/IL-6 ratio alone exhibited excellent discriminatory power (AUC 0.967) and maintained consistent performance on internal and temporal validation. The biological relevance of IL-10 in IVLBCL is further supported by cytokine profiling of lymphoma-associated hemophagocytic syndrome, in which elevated IL-10 was closely associated with B-cell lymphoma activity [27]. By incorporating this ratio alongside the three clinical variables, Model 2 achieved an AUC of 0.982, significantly surpassing Model 1 (DeLong test, *p* = 0.021). We therefore propose a two-step diagnostic strategy (Figure 5): Model 1 serves as an initial, widely applicable first step in hospitalized FUO patients; when positive, IL-10 and IL-6 are measured and Model 2 refines the risk estimate before invasive biopsy. In settings without cytokine testing, Model 1 alone remains a practical trigger for further investigation in this population. Critically, because neither model achieves perfect sensitivity, negative predictions must be interpreted with caution. For patients with a negative Model 1 result, standard FUO evaluation should continue, with periodic reassessment and a low threshold for measuring IL-10/IL-6 or performing random skin biopsy if clinical suspicion persists or new findings emerge. Similarly, for patients with a negative Model 2 result but high clinical suspicion, biopsy should still be considered. This safety-net pathway is essential to avoid dismissing treatable cases of IVLBCL, especially in patients with milder or atypical presentations.

The concept of a probability scoring system for IVLBCL is not new. Prior scoring systems have been proposed to identify patients with suspected IVLBCL for random skin biopsy, such as the Takigawa score [28] and the Tanaka score [29]. However, those systems were not specifically developed for patients presenting with FUO, and none incorporated the IL-10/IL-6 ratio, which has high diagnostic value for IVLBCL. Predictive models have also been developed for related conditions, such as EBER-positive lymphoma-associated hemophagocytic lymphohistiocytosis [30]. The present models therefore provide an FUO-specific approach that integrates this biomarker with readily available clinical variables. Accordingly, the novelty of our work should be understood as an FUO-specific diagnostic strategy rather than as the first IVLBCL scoring system overall.

The pattern of tissue sampling in our cohort reflects contemporary practice and supports the central role of random skin biopsy [31,32]. Most patients (58%) were ultimately diagnosed by random skin biopsy, with a positivity rate of 79.5%, consistent with recent Japanese series in which this technique has become the standard diagnostic procedure [6,8,15,25]. Bone marrow biopsy, although performed in the majority of patients, had a substantially lower yield (42.1%), while transbronchial lung biopsy was uniformly positive in the small subset of patients with overt pulmonary involvement. Critically, among patients predicted positive by Model 1 or Model 2, the random skin biopsy positivity rates reached 80%, indicating that the models can effectively select hospitalized patients who will most benefit from this procedure. These findings were reproduced in the external cohort, where random skin biopsy also demonstrated 100% positivity, confirming the robustness of the recommended diagnostic pathway.

Our results must be weighed against certain limitations. The training and temporal validation cohorts were derived from a single tertiary center, and the external case-only sensitivity evaluation cohort, although multicenter, included only IVLBCL cases without FUO controls, which precluded calculation of specificity and AUC in that setting. Consequently, the false-positive rate of Model 1 in unselected FUO populations remains unknown. Additionally, the absence of routine IL-10 and IL-6 measurement in external hospitals restricted external case-only sensitivity evaluation to Model 1; however, this limitation highlights the practical necessity of a purely clinical prediction tool. The wide confidence intervals for some odds ratios indicate that the estimates may be unstable due to the modest sample size, an inherent challenge in the study of an ultra-rare lymphoma. Although the present data support the use of these models in hospitalized patients with FUO, their applicability to outpatient or community settings is less clear. The proposed safety-net pathways may mitigate some of this uncertainty, but they rely on clinical judgment and may not be consistently applied across settings. Despite these caveats, the models address a genuine unmet need by offering, for the first time, a quantitative and easily implementable, FUO-specific strategy to raise early diagnostic suspicion of IVLBCL in hospitalized patients presenting with FUO. Prospective, multicenter studies with systematic cytokine measurement and appropriate FUO control groups are warranted to further refine and externally validate both models across broader patient populations.

## 5. Conclusions

In conclusion, Models 1 and 2 performed well in distinguishing IVLBCL from other causes of FUO in hospitalized patients. These models may assist physicians in the diagnostic workup of hospitalized patients with FUO. However, their utility in outpatient or community settings is less clear, and a negative result should not exclude IVLBCL. With the assistance of Model 1 and Model 2, actively conducting random skin biopsies in patients with a positive prediction can lead to a rapid diagnosis of IVLBCL. Prospective multicenter studies with appropriate FUO controls are needed to further validate these models and to determine their performance in broader, less selected populations.

## Figures and Tables

**Table 1:** Baseline characteristics of patients in the training cohort.

Variables	IVLBCL (n = 42)	FUO (n = 45)	*p* Value
Age, median (range), years	58.5 (32 to 76)	56 (21 to 73)	0.074
Sex, n (%)			
Male	23 (54.8%)	23 (51.1%)	0.831
Female	19 (45.2%)	22 (48.9%)	
Peripheral edema, n (%)	27 (64.3%)	4 (8.9%)	<0.001
Hypoxemia, n (%)	33 (78.6%)	5 (11.1%)	<0.001
Skin rash, n (%)	20 (47.6%)	16 (35.6%)	0.283
Neurological symptoms, n (%)	23 (54.8%)	8 (17.8%)	<0.001
Extremity weakness	11 (26.2%)	2 (4.4%)	
Cognitive impairment	6 (14.3%)	0	
Paraesthesia	4 (9.5%)	3 (6.7%)	
Slurred speech	3 (7.1%)	1 (2.2%)	
Disorder of consciousness	3 (7.1%)	5 (11.1%)	
Epilepsy	3 (7.1%)	0	
Visual disturbances	1 (2.4%)	0	
Hearing impairment	1 (2.4%)	0	
Hemophagocytic lymphohistiocytosis, n (%)	17 (40.5%)	5 (11.1%)	0.003
CT showed ILA, n (%)	27 (64.3%)	12 (26.7%)	0.001
IL-10 concentration, median (range), pg/mL	182.5 (0 to 1000)	0 (0 to 6.2)	<0.001
IL-10/IL-6, median (range)	11.0 (0 to 103.1)	0 (0 to 2.3)	<0.001

**Table 2:** Model 1 and Model 2 generated by multivariate logistic regression analysis. Model 1 comprised five clinical variables: peripheral edema, hypoxemia, neurological symptoms, HLH, and ILA. Model 2 incorporated the IL-10/IL-6 ratio with three clinical variables: peripheral edema, hypoxemia, and neurological symptoms.

Variables	Coefficient	OR (95% CI)	*p* Value
Model 1			
Intercept	−2.479	0.084 (0.023–0.231)	<0.001
Peripheral edema	1.677	5.348 (1.011–32.393)	0.053
Hypoxemia	2.592	13.354 (3.041–74.551)	0.001
Neurological symptoms	1.146	3.145 (0.724–14.639)	0.129
HLH	0.896	2.449 (0.366–15.960)	0.345
CT showed ILA	0.403	1.497 (0.287–7.050)	0.612
Model 2			
Intercept	−5.119	0.006 (0.000–0.054)	<0.001
Peripheral edema	1.488	4.428 (0.118–217.603)	0.412
Hypoxemia	3.450	31.504 (2.046–1797.170)	0.029
Neurological symptoms	0.027	1.028 (0.019–29.296)	0.988
IL-10/IL-6	2.219	9.195 (2.619–70.708)	0.006

**Table 3:** Differential diagnostic efficiency of IL-10/IL-6 concentration ratio, Model 1 and Model 2. The cutoff values were determined by calculating the point on the ROC curve that maximized the Youden index (sensitivity + specificity − 1). AUC was generated by ROC analysis. Std, standard error of the AUC estimated by the DeLong method. AUC, area under curve; ROC, receiver operating characteristic.

Parameter	Cut-Off Value	Std	AUC (95% CI)	Sensitivity	Specificity
IL-10/IL-6	0.450	0.018	0.967 (0.931–1.000)	97.6%	86.7%
Model 1	0.296	0.033	0.916 (0.851–0.981)	90.5%	84.4%
Model 2	0.576	0.018	0.982 (0.947–1.000)	97.6%	100.0%

**Table 4:** The pathological diagnosis of IVLBCL.

Biopsy Type	Random Skin Biopsy	Bone Marrow Biopsy	TBLB
Patients who received biopsy, n (% of all patients)	44 (73.3%)	57 (95.0%)	8 (13.3%)
Patients diagnosed by biopsy, n (% of all patients)	35 (58.3%)	24 (40.0%)	8 (13.3%)
Positive rate	79.5%	42.1%	100%

## Data Availability

The datasets used and/or analyzed during the current study are provided in Supplementary File S2, which is accessible alongside this article.

## Supplementary Materials

The following supporting information can be downloaded at: https://www.mdpi.com/article/10.3390/diagnostics16172768/s1, Supplementary File S1: IVLBCL_R_verification_code; Supplementary File S2: IVLBCL_revised_supplementary data.

## Abbreviations

The following abbreviations are used in this manuscript:

AUC	Area under the curve
CI	Confidence interval
CT	Computed tomography
FUO	Fever of unknown origin
HLH	Hemophagocytic lymphohistiocytosis
ILA	Interstitial lung abnormalities
IL-6	Interleukin-6
IL-10	Interleukin-10
IVLBCL	Intravascular large B-cell lymphoma
OR	Odds ratio
PaO_2_	Arterial oxygen partial pressure
ROC	Receiver operating characteristic
TBLB	Transbronchial lung biopsy
